# Modulation of microRNAs and claudin-7 in Caco-2 cell line treated with *Blastocystis* sp., subtype 3 soluble total antigen

**DOI:** 10.1186/s12866-022-02528-8

**Published:** 2022-04-22

**Authors:** Hanieh Mohammad Rahimi, Abbas Yadegar, Hamid Asadzadeh Aghdaei, Hamed Mirjalali, Mohammad Reza Zali

**Affiliations:** 1 Foodborne and Waterborne Diseases Research Center, Research Institute for Gastroenterology and Liver Diseases, Shahid Beheshti University of Medical Sciences, Tehran, Iran; 2 Basic and Molecular Epidemiology of Gastrointestinal Disorders Research Center, Research Institute for Gastroenterology and Liver Diseases, Shahid Beheshti University of Medical Sciences, Tehran, Iran; 3 Gastroenterology and Liver Diseases Research Center, Research Institute for Gastroenterology and Liver Diseases, Shahid Beheshti University of Medical Sciences, Tehran, Iran

**Keywords:** *Blastocystis* sp., ST3, MicroRNA, Intestinal permeability, Claudin-7, Inflammation

## Abstract

**Background:**

*Blastocystis* sp., is a eukaryote of the large intestine, which is reported from almost all countries. The pathogenesis of this protist is not clear. The current study aimed to analyze the effects of *Blastocystis* sp., ST3 soluble total antigen (B3STA) on the microRNAs (miRNAs) involved in the gut permeability and also pro-inflammatory cytokines, occludin, and claudin-7.

**Methods:**

*Blastocystis* sp., ST3 isolated from stool sample was purified, and its soluble total antigen was extracted using freeze and thawing. The Caco-2 cell line was treated with B3STA for 24 h and the expression levels of mir-16, mir-21, mir-29a, mir-223, and mir-874 were analyzed. In addition, the expression levels of *il-8*, *il-15*, *occludin*, and *claudin-7* genes were assessed.

**Results:**

B3STA significantly upregulated the expression of mir-223, and mir-874, and downregulated mir-29a. The expression of mir-16 and mir-21 was not significant. In addition, the expression of *il-8* and *il-15* was not significant. B3STA significantly decreased the expression level of *claudin-7* (*P*-value < 0.0001), but the expression of *occludin* was not significant. Our results showed significant correlation between all studied miRNAs, except mir-29a, with downregulation of *claudin-7*.

**Conclusions:**

This is the first study investigating the effects of *Blastocystis* sp., ST3 isolated from symptomatic subjects on the expression levels of miRNAs involved in the gut permeability. Our results demonstrated that B3STA may change miRNA expression, which are involved in the gut barrier integrity, and downregulates *claudin-7*, which is known as sealing factor.

## Background

*Blastocystis* sp., is a single cell protist which colonizes the large intestine of humans and a wide range of animals [1, 2]. This protist is transmitted via fecal-oral rout, contaminated food and water, and close contact to animals [3, 4]. This protist is one of the most prevalent eukaryotes, which its prevalence rate reaches up to even 100% [5].

Based on the phylogenetic and molecular analyses of a signature region through the small subunit ribosomal RNA (SSU rRNA) gene of *Blastocystis* sp., at least 22 distinct subtypes have been reported from humans and animals [6]. From these subtypes, subtype (ST) 3 thought to be the most prevalent subtypes with wide distribution all over the world [4]. Most of cases that carry *Blastocystis* sp., in their intestine do not complain from specific symptoms [7–9]; however, available evidence has linked *Blastocystis* sp., colonization with some clinical manifestations. Accordingly, most of studies have associated the presence of ST1 and ST3 with some clinical manifestations such as gastrointestinal disorders and urticarial [10–12].

It was documented that *Blastocystis* sp., may affect its hosts via manipulation of either the gut microbiome or the host’s immune system [13–15]. Although it is controversially [13, 16–18], *Blastocystis* sp., is suggested to increase the gut microbiota diversity and to be a healthy gut indicator [19–22]. Nevertheless, the release of proteases from *Blastocysis* sp., supposes to disrupt tight junctions (TJ) or destroy the secretory immunoglobulin A (sIgA) [23–26]. A recent study evaluated protease activity and the effects of *Blastocystis* sp., subtypes 1–3 and 6 isolated from symptomatic and asymptomatic subjects in HT-29 cell line, and claimed higher protease activity of *Blastocystis* sp. isolated from symptomatic carriers [27]. They showed a significant higher protease activity of *Blastocystis* sp., ST3 isolated from symptomatic subjects compared to those isolated from asymptomatic individuals [27]. However, a lot of aspects remain unclear such as how *Blastocystis* sp., communicate with its hosts.

MicroRNAs (miRNAs) are 17–25 nucleotides, non-coding RNA fragment, which was firstly discovered in *Caenorhabditis elegans*. From 1993, which the first miRNA, *lin-4*, was described, new miRNAs are still being characterized. MiRNAs mostly interact with 3′ UTR of target messenger RNAs (mRNAs) to regulate their expression [28, 29]. Although there is evidence of communication between parasites and their hosts via miRNAs, there is no study describing the role of *Blastocystis* sp., on the expression of miRNAs involved in the gastrointestinal homeostasis. The current study aimed to investigate the effects of soluble total antigen (STA) of *Blastocystis* sp., ST3 on the expression changes of miRNAs: mir-16, mir-21, mir-29a, mir-223, and mir-874, which play roles in the integrity of the intestine, in the Caco2 cell line.

## Methods

### Ethical approval

No human or animal tissues were analyzed in this study. The study was approved by the ethics committee/institutional review board of the Research Institute for Gastroenterology and Liver Diseases, Shahid Beheshti University of Medical Sciences, Tehran, Iran.

All experimental protocols were approved by the Research Institute for Gastroenterology and Liver Diseases and all procedures of this study were in accordance with the ethical standards (IR.SBMU.RIGLD.REC.1398.048) released by the Ethical Review Committee of the Research Institute for Gastroenterology and Liver Diseases, Shahid Beheshti University of Medical Sciences, Tehran, Iran. In addition, all methods were carried out in accordance with relevant guidelines and regulations.

### *Blastocystis* isolate

In the current study, *Blastocystis* sp., ST3 was from stool sample of a symptomatic subject from our previous study [7, 27]. Briefly, *Blastocystis* sp., was isolated from stool samples, which were cultured in Dulbecco’s Modified Eagle Medium (DMEM) (Gibco, Thermo Fisher Scientific, MA, USA) containing penicillin-streptomycin (Sigma, USA), (1000-unit penicillin and 4 mg/mL streptomycin) supplemented with 10% heat-inactivated fetal bovine serum (FBS, Sigma-Aldrich, USA), and were incubated in an anaerobic condition at 37 °C. The studied *Blastocystis* sp., ST3 was purified using Ficoll gradient (Ficoll-Paque™ PREMIUM) and several consecutive subculture in combination with antibiotic cocktail for about six months [27]. To characterize the subtype of isolated *Blastocystis* sp., the barcoding fragment was amplified using specific primers and sequenced [30]. To confirm that *Blastocystis* sp., is purified, consecutive cultivation of the protist was performed accompanied with a mixture of active antibiotics (4000 mg/ml of ampicillin, 1000 mg/ml of streptomycin, and 1000 units of penicillin together with amphotericin B (50 mg/mL) to eliminate yeasts or filamentous fungi), which were determined using antibiogram. Details of purification are mentioned elsewhere [27].

### *Blastocystis* sp. ST3 soluble total antigen (B3STA)

To prepare soluble total antigen from purified *Blastocystis* sp., 1 × 10^5^ parasites/mL of the parasite was washed three times in PBS at 300×*g* for 5 min at 4 °C and counted with Neubauer’s improved cell counting chamber (perci color HBG; Germany). In order to prepare the B3STA, three freeze-thaw cycles in liquid nitrogen and a 37 °C water bath were employed and the resultant subject was filtered using polyethersulfone (PES) filters with 0.22 μm pore size to eliminate probable remained bacteria [27].

### Cell culture

The human colorectal adenocarcinoma cell line (Caco-2; ATCC HTB-37) was cultivated in a 25-cm^2^ culture flask (Cell culture Flask, SPL, Korea) supplemented with 5 mL of high-glucose DMEM medium (DMEM High Glucose, Biosera), 5% (v/v) heat-inactivated FBS, 2 mM L-glutamine, and 1% antibiotic-antimycotic agents (penicillin: 100 U/ml, streptomycin: 100 mg/ml Sigma-Aldrich, USA). Cultivated Caco-2 cells were incubated in 5% CO_2_ and 100% humidity at 37 °C. Upon 70–80% confluency, the cells were washed with sterile PBS (pH = 7), detached using 0.25% trypsin-EDTA (Gibco, USA), and the number of alive cells were counted by 0.025% (w/v) trypan blue solution (Gibco, USA) and Neubauer’s improved cell counting chamber*.*

### Co-incubation of B3STA with Caco-2 cell line

For this purpose, 1 × 10^5^ Caco-2 cells were counted and seeded in each well of a six-well plate. The plate was incubated in 5% CO_2_ at 37 °C overnight. After 70–80% confluency, the B3STA prepared from 10^5^ of *Blastocystis* sp. ST3 was added to the sample well. In addition, 20 ng/mL LPS (Santa Cruz Biotechnology Cat No. sc-3535) were used to compare the induction pattern to the B3STA. A well full of Caco-2 cell without any treatment either by B3STA or LPS, was considered as control group. All groups were in duplicate and evaluated 24 h after exposure.

### MicroRNA selection and primer designing

In order to evaluate the expression level of miRNAs: mir-16, mir-21, mir-29a, mir-223, and mir-874, mature sequences of human miRNAs were selected from the miRBase database (https://www.mirbase.org/) according following accession numbers: MIMAT0000069, MIMAT0000076, MIMAT0000086, MIMAT0000280, and MIMAT0004911, respectively. The primer designing was performed based on stem-loop and regarding the protocol, which was previously explained [31] (Table 1). The stem-loop reverse transcriptase (RT) and real-time PCR primers provide higher specificity and efficacy.

**Table 1:** Stem-loop RT primers designed for each studied miRNA and its forward real-time PCR primer

Gene name	Primers		Tm (°C)
*Hsa-16-5p*	Real-time	ACACTCCAGATGGGTAGCAGCACGTAA	60
	RT	CTCAACTGGTGTCGTGGAGTCGGCAATTCAGTTGAGCGCCAATA	60
*Hsa -29a-3p*	Real-time	ACTCTCGAGCACTGTAGCACCATCTGAA	60
	RT	CTCAACTGGTGTCGTGGAGTCGGCAATTCAGTTGAGTAACCGAT	60
*Hsa −21-5p*	Real-time	ACACTCCAACAGGGTAGCTTATCAGACT	60
	RT	CTCAACTGGTGTCGTGGAGTCGGCAATTCAGTTGAGTCAACATC	60
*Hsa −223-3p*	Real-time	AGTCTCCAGCAGGGTGTCAGTTTGTCAA	60
	RT	CTCAACTGGTGTCGTGGAGTCGGCAATTCAGTTGAGTGGGGTAT	60
*Hsa −874-3p*	Real-time	ACACTCCTGCTGCGCTGCCCTGGCCCGA	60
	RT	CTCAACTGGTGTCGTGGAGTCGGCAATTCAGTTGAGTCGGTCCC	60
*U6*	Real-time	ACACTCCATCTGGGTCGTGAAGCGTTC	60
	RT	CTCAACTGGTGTCGTGGAGTCGGCAATTCAGTTGAGAAAAATATG	60
Universal reverse	Real-time	TGGTGTCGTGGAGTCGGCAATTCAGTTG	60

Note: *Hsa Homo sapiens*, *RT* reverse transcriptase, *Tm* melting temperature

### RNA extraction, cDNA synthesis, and quantitative real-time PCR

Total RNA was extracted using total RNA purification mini kit (YTA, Tehran, Iran). In order to adjust the RNA concentration before complementary DNA (cDNA) synthesis, the concentration of extracted RNAs was determined by a NanoDrop (NanoDrop Technologies, USA) apparatus. The cDNA synthesis specific for each miRNA was constructed using cDNA synthesis kit (YTA, Tehran, Iran) as explained previously [31]. During cDNA synthesis, stem–loop RT primers are used instead of conventional RT primers, and bind to the 3′ end of miRNA to increase the length of target miRNA [31].

To amplify and quantify targeted miRNA using real-time PCR, miRNA-specific forward primer and a universal reverse primer are used. Forward primers for real-time PCR are designed to bind to the 5′ end of miRNA, which was constructed using stem-loop RT primers, as tailed forward primer, and to increase the melting temperature (Tm) of target miRNA sequence [31].

To analyze the effects of B3STA on the inflammatory biomarkers and TJ, the expression levels of IL-8, IL-15, occludin, and claudin-7 were evaluated (Table 2). Relative expression of the miRNAs in treated and untreated cells were determined by quantitative (q) real-time PCR using Rotor-Gene Q (Qiagen, Germany) in a 20 μL reaction mixture containing 10 μL SYBR Green qPCR master mix 2X (Ampliqon, Denmark), 5 ρM of each primer, and 2 μL of constructed cDNA as template. The amplification conditions for miRNAs were adjusted with previously released protocol [31]. Real-time PCR for inflammatory markers and TJ was performed using Rotor-Gene Q (Qiagen, Germany) thermocycler in a 20 μL reaction mixture containing 10 μl SYBR Green qPCR Master Mix 2X (Ampliqon, Denmark), 5 μM of primers, and 2 µL of cDNA under conditions: initial denaturation 95 °C for 10 min, followed by denaturation at 95 °C for 20 s, annealing at 58–63 °C for 30 s, and extension at 72 °C for 20 s.

**Table 2:** Employed primers to study the expression of cytokines and tight junction’s genes.

Genes	Sense primer (5′-3′)	Antisense primer (5′-3′)	Tm (°C)	Refs
*il-8*	TGGCTCTCTTGGCAGCCTTC	TGCACCCAGTTTTCCTTGGG	60	[32]
*il-15*	TGTCTTCATTTTGGGCTGTTTCA	GAATACTTGCATCTCCGGACTC	60	[33] with some modifications
*claudin-7*	AGCTGCAAAATGTACGACTCG	GGAGACCACCATTAGGGCTC	57	[34]
*occludin*	CCACGCCGGTTCCTGAAGTGG	TCACAGGACTCGCCGCCAGT	63	[35]
*Bbeta actin*	ATGTGGCCGAGGACTTTGATT	AGTGGGGTGGCTTTTAGGATG	60	[36]

Note: *Il* interleukin, *Tm* melting temperature

To avoid from non-specific amplification, melting curve analysis was employed for each run. Subsequently, the relative quantification (RQ) of each miRNA relative to U6 snRNA [37] and inflammatory markers and TJ relative to beta-actin (BACT) was calculated using 2^-∆∆CT^ incorporated in relative expression software tool (REST). All tests were performed in duplicate.

### Statistical analysis

Student’s t-test was applied to analyze the real-time PCR data. *P* value < 0.05 were considered statistically significant. Statistical analysis was performed using GraphPad Prism software version 8.3.0.538.

## Results

### Relative expression of miRNAs

**Fig. 1:**
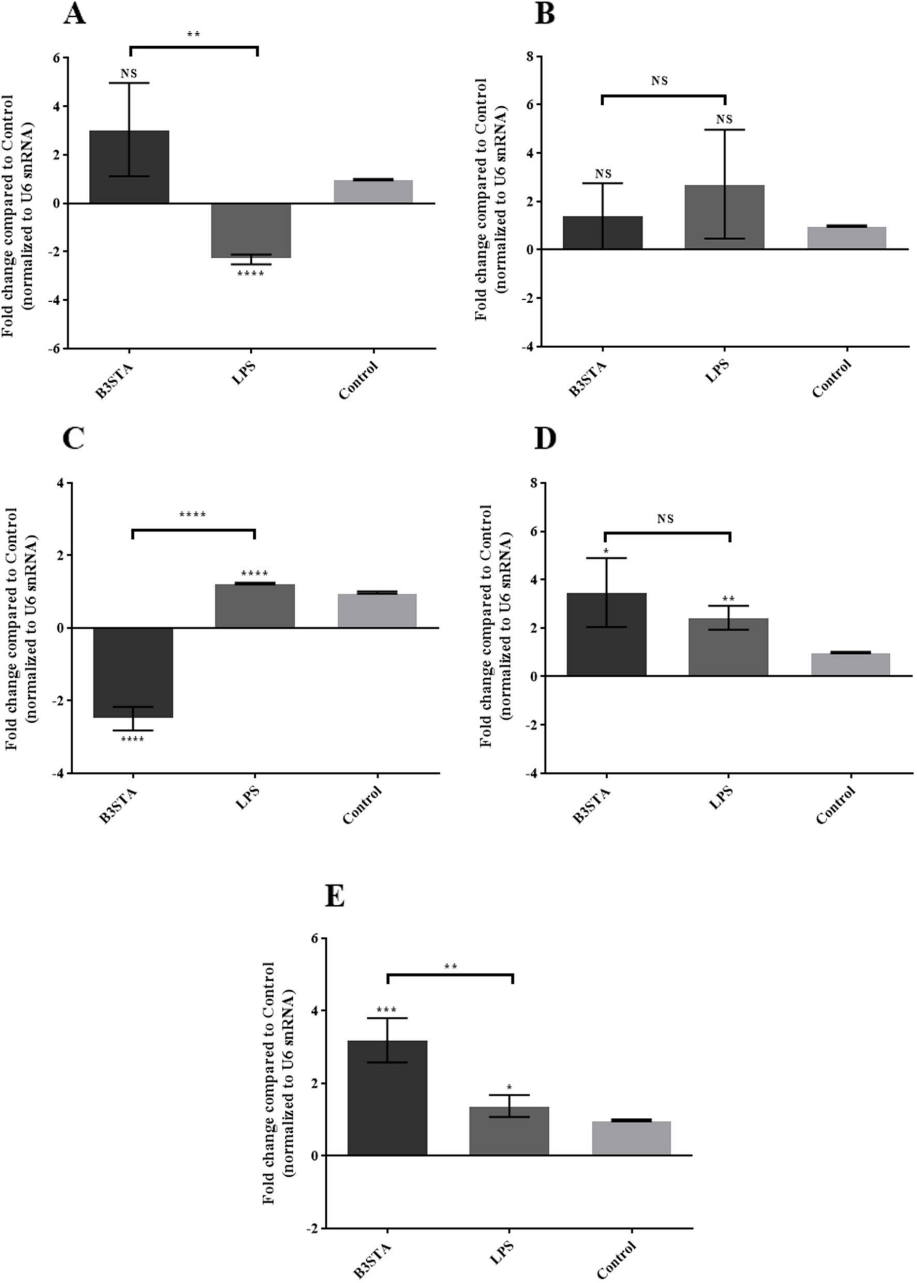
The expression levels of (**A)** mir-16, (**B)** mir-21, (**C)** mir-29a, (**D)** mir-223, and (**E)** mir-874 in the Caco-2 cell line co-incubated with B3STA, isolated from symptomatic carrier, 24 h after exposure. Accordingly, the B3STA significantly downregulated mir-29a, and upregulated mir-223 and mir-874. In addition, LPS significantly downregulated mir-16, while upregulated mir-29a, mir-223, and mir-874. Comparison between LPS and B3STA was statistically significant only in mir-16, mir-29a, and mir-874. * *P* value < 0.05; ** *P* value < 0.01; *** *P* value < 0.001; **** *P* value < 0.0001. Comparisons were carried out using the Student’s t-test. Mir: microRNA; Caco-2: human colon carcinoma; B3STA: *Blastocystis* sp., ST3 soluble total antigen; LPS: lipopolysaccharide; NS: not significant

The B3STA did not significantly changes mir-16 3 (.041 folds; *P*-value *=* 0.0754), but LPS significantly downregulated the levels of mir-16 for 2.328 folds in the Caco-2 cell line (*P*-value *<* 0.0001). The comparison of the expression levels of mir-16 between the B3STA and LPS showed significant difference (*P*-value *=* 0.0014) (Fig. 1A).

The expression level of mir-21 was not significantly changed with the B3STA (1.387 folds; *P*-value *=* 0.567), and LPS (2.716 folds; *P*-value *=* 0.174) (Fig. 1B). The B3STA significantly downregulated the expression levels of mir-29 (2.497 folds; *P*-value < 0.0001), while LPS significantly upregulated mir-29 (1.224 folds; *P*-value < 0.0001). Indeed, the comparison of the expression of mir-29 between the B3STA and LPS was statistically significant (*P*-value < 0.0001) (Fig. 1C). Our result showed that the B3STA and LPS significantly increased the expression levels of mir-223 (3.463 folds; *P*-value = 0.0128) and (2.425 folds; *P*-value = 0.0011), respectively (Fig. 1D). Finally, the expression of mir-874 was evaluated in Caco-2 cell line sensed by the B3STA that the results showed statistically significant overexpression in the cells, which were treated with the B3STA (3.186 folds; *P*-value = 0.0004) and LPS (1.37 folds; *P*-value = 0.0391). The comparison of the expression of mir-874 between the B3STA and LPS was statistically significant (*P*-value = 0.0018) (Fig. 1E).

### Relative expression of inflammatory and TJ markers

**Fig. 2:**
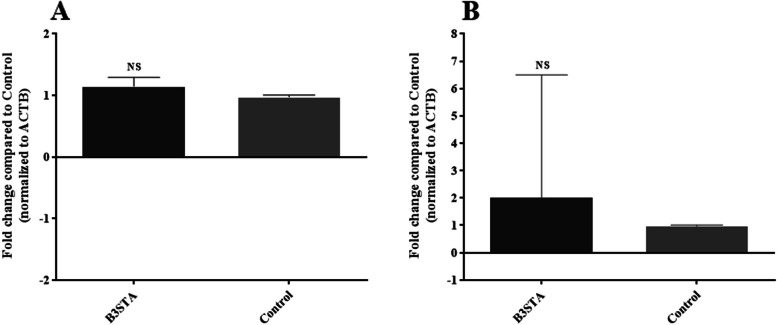
The expression levels of (**A) ***il-8* and (**B) ***il-15* genes in the Caco-2 cell line co-incubated with B3STA, 24 h after exposure. Our analysis indicated that changes in both cytokines were not statistically significant. Comparisons were carried out using the Student’s t-test. IL: interleukin; Caco-2: human colon carcinoma; B3STA: *Blastocystis* sp., ST3 soluble total antigen

**Fig. 3:**
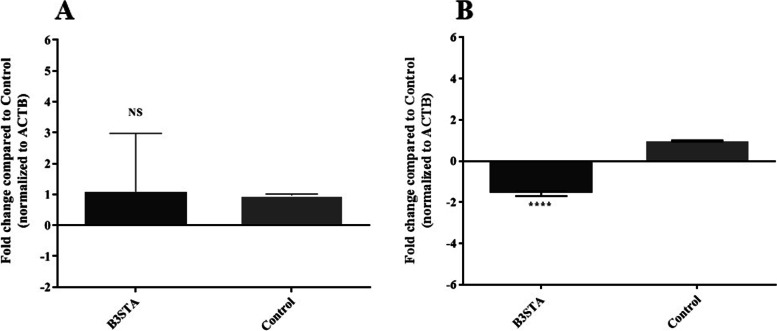
The expression levels of (**A)** *occludin* and (**B) ***claudin-7* genes in the Caco-2 cell line co-incubated with B3STA, 24 h after exposure. Statistically significant downregulation was seen in *claudin-7*. Claudin-7 is known as sealing factor and plays important role in reducing the gut permeability. **** *P* value < 0.0001. Caco-2: human colon carcinoma; B3STA: *Blastocystis* sp., ST3 soluble total antigen. Comparisons were carried out using the Student’s t-test

The results of relative expression showed no statistically significant changes in *il-8* (1.148 folds; *P*-value = 0.0584) and *il-15 *genes (2.017 folds; *P*-value = 0.7737) (Fig. 2). The B3STA significantly downregulated claudin-7 (1.582 folds; *P*-value < 0.0001), but did not induce significant changes in occludin (Fig. 3).

### Correlation between miRNAs and expression of inflammatory and TJ markers

The correlation between the expression of *il-8* and *il-15* genes with investigated miRNAs was assessed and showed that only *il-8* had a significant correlation with mir-29a (*P*-value = 0.007) and mir-874 (*P*-value = 0.011), while the correlation between *il-8* and *il-15* and all other miRNAs was not statistically significant. The correlation between the expression of *occludin* and miRNAs was not statistically significant. In addition, except mir-29a, all studied miRNAs were significantly correlated with elevated level of *claudin-7* (Table 3).

**Table 3:** The statistical correlation of each miRNA with studied cytokines and tight junctions

Tight Junctions				Cytokines				miRNA
Claudin-7		Occludin		IL-15		IL-8		
***P***-value	Sig.	***P***-value	Sig.	***P***-value	Sig.	***P***-value	Sig.	
0.032	Yes	0.305	No	0.693	No	0.26	No	**mir-16**
0.044	Yes	0.832	No	0.788	No	0.826	No	**mir-21**
0.093	No	0.12	No	0.291	No	0.007	Yes	**mir-29a**
0.009	Yes	0.152	No	0.549	No	0.096	No	**mir-223**
0	Yes	0.089	No	0.589	No	0.011	Yes	**mir-874**

Note: *miRNA and mir* microRNA, *IL* interleukin, *Sig* significant

## Discussion

In the current study, we employed B3STA, which was obtained from a symptomatic subjects from our previous study [27]. The main reason for choosing this isolate was to investigate the effects of a clinically isolated *Blastocystis* sp., because it is documented that continuous cultivation of eukaryotes in axenic conditions may affect their physiological and pathogenic features [38, 39].

Focuses on the interaction between parasites and their hosts have pointed out the critical role of host or parasite originated miRNAs in orchestrating the immune responses and pathogenesis of parasites [40–42]. *Blastocystis* sp., is a prevalent protist, which its pathogenicity is still unclear. Nevertheless, the number of studies, which are describing pathogenic role for *Blastocystis* sp., are increasing. It was documented that *Blastocystis* sp., is not only able to dysregulate immune responses during colonization in the intestine [26, 43], but also affects the permeability of the gut [24, 44], both using its proteases. For example, Puthia et al., (2006) [45] observed that *B. ratti* induced apoptosis and increased the permeability of the gut epithelium. Additionally, Mirza et al., (2012) [24] demonstrated that *Blastocystis* sp., is able to manipulate cell permeability, transepithelial resistance, and phosphorylation of myosin light chain via a rho kinase (ROCK)-dependent manner. Another study by the same team, which was performed in Caco-2 cell line, suggested that ST7 changed the permeability and tight junction localization, which that led to disruption of the intestinal barrier [46].

The intestinal barrier plays crucial role in keeping homeostasis of the gut and protecting from translocation of the gut contents into the lower layers, such as mucus and the circulation system [47]. Therefore, an intestinal barrier with impaired functions has been linked to a broad spectrum of immunological disorders in gastrointestinal system [47, 48]. Tight junction proteins keep the integrity of intestinal barriers, and disruption of these proteins increases paracellular and transcellular permeability [47]. Claudins, occludin, and zonula occludens are the most important TJ proteins that are involved in maintenance of the intestinal barriers [48–50]. In the current study the effect of B3STA on occludin was almost without change, while it significantly decreased the expression level of claudin-7*claudin-7*. Claudin-7 in humans is observed in the large intestine [51], where is colonized by *Blastocystis* sp. Although controversially [49], claudin-7 is categorized among pore-sealing groups of claudins and are responsible for sealing junctions and reducing permeability [52].

The gut barrier integrity and permeability are also suggested to be regulated by miRNAs [52, 53] (Table 4). As results, B3STA significantly upregulated mir-223 and mir-874, and downregulated mir-29a. It was documented that mir-16 may be involved in the intestinal barrier dysfunction [78]. Although it was claimed that the level of mir-16 was downregulated in irritable bowel syndrome (IBS) diarrhea predominant patients [78], it was shown that in IBD patients the expression level of mir-16 was elevated in inflammatory bowel diseases (IBD) patients [79], and therefore, inhibiting of mir-16 could be an alternative therapeutic strategy [80]. Similar to mir-16, mir-21 seems to be associated with the impaired functions of intestinal barrier. Zhang et al., (2015) [61] assessed mir-21 in Caco-2 cell line and showed a significantly increased expression of this miRNA in the intestinal TJ barrier defect model, which was associated with overexpression of IL-8. In this line, it was claimed that mir-21 is correlated with ischemia reperfusion [81], flare up in IBD [82–84], and proliferation and invasion of colon adenocarcinoma [85], which all are related to the intestinal barrier dysfunction. As a result, B3STA downregulated mir-29. The mir-29 family thought to be involved in development of fibrosis, particularly in IBD [86–88]. On the other hand, in a clinical study in IBS patients, it was proposed that overexpression of mir-29a could be related to the glutamine synthesis and gut permeability [89]. Actually, mir-29a increases the gut permeability via controlling glutamine synthesis [89]. In this line, an experimental study performed in intestinal epithelial cell line (IPEC-1) suggested that inhibiting mir-29a was related to improvement of the monolayer integrity [87]. Our results also showed that B3STA increased the expression levels of mir-223 and mir-874. It was documented that mir-223 is associated with inflammation through the intestine tissue [90–92]. In addition, Li et al., (2020) suggested that mir-223-enriched mast cell-derived exosomes inhibited TJ proteins and destroyed intestinal barrier functions [92]. Notable, it was demonstrated that mir-874 induced paracellular permeability and intestinal barrier dysfunction via changes in expression of aquaporin 3 (AQP3) protein [73, 93]. Many studies reported a low prevalence rate of *Blastocystis* sp., colonization in IBD patients [13, 94–96], and a high prevalence of the protist in IBS patients [97–100]. However, it is though that the colonization of *Blastocystis* sp., probably is an indicator for healthy gut [19, 21, 22, 94].

**Table 4:** Targets and biological functions of studied miRNAs in intestinal permeability

miRNAs	Targetd factors/pathways	Biological functions	Attributed diseases	Ref
**mir-16**	Claludin-2 Cingulin P38 MAP kinase P53 phosphorylation	Barrier function dysregulation Apoptosis	Ulcerative colitis (UC) Crohn’s disease (CD) IBS Colorectal adenocarcinoma	[54–60]
**mir-21**	Rho-associated protein kinase 1(ROCK1) PTEN/PI3K/AKT Signaling PTEN/ PDCD4/ROCK1 Tight junctions (occludin) ADP ribosylation factor 4 (ARF4)	Regulates tight junction proteins Protects intestinal barrier from dysfunction Regulates intestinal tight junction Permeability of the intestinal barrier Inflammation	IBD	[54, 56–58, 61–64]
**mir-29a**	Vascular endothelial growth factor A (VEGF-A) Telomerase Reverse Transcriptase (TERT) Integrin B1 (ITGB1) Roundabout guidance receptor 1 (ROBO 1) APC domain containing 2 (p42.3 or SAPCD2) Cyclin-dependent kinase (CDK) 2, 4, and 6 Vascular endothelial growth factor (VEGF) Tight junction proteins	Inhibiting metastasis related genes Inhibiting cell proliferation of GC cells Suppressed angiogenesis Apoptosis Intestinal barrier integrity	UC Gastric cancer (GC) CD	[54, 59, 65–70]
**mir-223**	RAS p21 GTPase-activating protein 1 (RASA1) Claudin-8 (CLDN8)	Proliferation Pro-inflammatory Mediates the cross-talk between the IL23 pathway and the intestinal barrier in IBD	Non-inflamed UC Non-inflamed CD IBD	[57, 59, 71, 72]
**mir-874**	Aquaporins (AQPs) Signal transducer and activator of transcription (STAT)3 YAP/TAZ signaling X-linked inhibitor of apoptosis protein (XIAP)	Promotes intestinal barrier dysfunction Anti-oncomir Drug resistance	Intestinal ischemic injury Esophageal squamous cell carcinoma (ESCC) Colorectal cancer (CRC)	[73–77]

Note: *miRNA and mir* microRNA, *UC* ulcerative colitis, *CD* Crohn’s disease, *IBS* irritable bowel syndrome, *IBD* inflammatory bowel diseases, *GC* gastric cancer

Collectively, the significant expression changes of mir-29a, mir-223, and mir-874 in Caco-2 cell line treated with B3STA together with the significant correlation between overexpression of mir-223, and mir-874 with claudin-7 suggest a cross-talk between *Blastocystis* sp., and its host that should be scrutinized not only using in vitro models, but also by in vivo studies.

The main limitation of the current study was investigation of the effects of *Blastocystis* sp., in cell culture. Although in vitro investigations are important part of molecular biology studies, inferring the role of *Blastocystis* sp., in manipulation of the gut permeability in humans needs more experimental and clinical surveys.

## Conclusion

Our results also showed that B3STA isolated from symptomatic carrier, is able to change miRNA expression of mir-29a, mir-223, and mir-874, which are involved in the gut barrier integrity. In addition, *Blastocystis* sp., can downregulates claudin-7, which is known as sealing factor. This study provides a clue about the role of miRNAs on pathogenesis of *Blastocystis* sp., but further studies, in vitro and in vivo, are needed to clear correlation between *Blastocystis* sp., and expression changes of host’s miRNAs.

## Data Availability

Generated data including figures and tables were not submitted elsewhere and are included in the article. In this study, DNA or RNA sequences were not generated to be submitted in relevant databases.
